# Multilocus analysis of *Gallid herpesvirus* 1 in layer chickens in Iraq

**DOI:** 10.14202/vetworld.2020.170-176

**Published:** 2020-01-24

**Authors:** Mohammed Hamzah Abdulkadhim Al-Saadi

**Affiliations:** Department of Veterinary Internal Medicine, College of Veterinary Medicine, University of Al-Qadisiyah, Al Diwaniyah, Iraq

**Keywords:** *Gallid herpesvirus* 1, infectious laryngotracheitis virus, infectious laryngotracheitis, polymerase chain reaction

## Abstract

**Background and Aim:**

Infectious laryngotracheitis virus (ILTV) causes a highly pathogenic respiratory disease that affects poultry. It is also known as *Gallid herpesvirus* 1. ILT prophylaxis measures often include using live attenuated vaccines. The live attenuated vaccine can, however, lead to the formation of new strains of ILTV as a result of vaccine reversion and recombination with field strains. Therefore, this study was performed to explore the multilocus variation of ILTV strains of field and vaccine origin. Samples were tested from two distinctive geographical areas in Iraq as little is known about the ILTV genetic diversity within these areas.

**Materials and Methods:**

The polymerase chain reaction method was utilized to generate sequencing templates of six highly polymorphic genes, including UL54, UL52, gB, ICP18.5, ICP4, and gJ in the layer chicken sample (n=15). The Western blotting technique was also employed to detect and estimate the native molecular weight of gE.

**Results:**

The results revealed an important degree of genetic relatedness between the field and vaccine strains across all genes. In addition, gE was found to be expressed natively at 49 kDa.

**Conclusion:**

The findings of this study may be used to improve the production process of the vaccine for more effective ILT prophylaxis and could further the understanding of epidemiologists and immunologists to better control ILT in the future.

## Introduction

Infectious laryngotracheitis (ILT) is an avian respiratory disease caused by *Gallid herpesvirus* 1 (GaHV-1). It is an alpha herpesvirus that belongs to *Iltovirus* genus [1]. The disease is globally distributed and results in economic losses; due to a drop in egg production, reduced weight gain, and mortality [2]. The genome of ILT virus (ILTV) is large; approximately 149 kb of double-stranded DNA arranged in 77 open reading frames [3]. There are many strains of ILTV with more than 99% homology of the genome, making epidemiological studies difficult to conduct [4]. ILTV has latency-associated transcripts that are highly conserved in all viruses belonging to the Herpesviridae family [5]. These transcripts are highly expressed during the latent phase of the disease and act to suppress infection reactivation by reducing general viral genomic expression. This process plays a leading role in the latency phase of ILT and allows the virus to persist inside the host cells for long periods of time [6]. Viral genomic recombination with host cells is an evolutionary host evasion mechanism that allows the virus to evade detection by the host’s immune system. This is a characteristic feature of herpesviruses [7].

Live attenuated vaccines can be produced from chicken embryo origin (CEO) or tissue culture origin [8]. However, these types of vaccines frequently revert and recombine with natural strains to form new vaccinal laryngotracheitis (VLT) strains [9]. Thus, we hypothesized that ILTV outbreaks in Iraq are caused by genetic interference between field and vaccine strains. Understanding the genetic interference of ILTV strains is essential to control the disease.

Restriction fragment length polymorphism (RFLP) has been used to discriminate ILTV strains. However, it is relatively expensive and labor-intensive in comparison with real-time polymerase chain reaction (PCR), but the latter and former methods could be combined to provide a better result [10]. Recently, multilocus sequence typing by the PCR technique has been used to distinguish between ILTV strains [11]. This technique was therefore adopted in the current study to demonstrate the phylogenetic relationship between ILTV strains by sequencing six different genomic regions, including UL54, UL52, gB, ICP18.5, ICP4, and gJ. This study was conducted to investigate the genetic variation of GaHV-1 by multilocus PCR sequencing. This technique has the advantage of determining allelic discrepancies accurately and rapidly [11]. Therefore, we employed this technique to amplify six genomic regions followed by sequencing.

Therefore, this study was performed to explore the multilocus variation of ILTV strains of field and vaccine origin. Samples were tested from two distinctive geographical areas in Iraq as little is known about the ILTV genetic diversity within these areas.

## Materials and Methods

### Ethical approval

The Ethics Review Board of Al-Qadisiyah College of Agriculture and College of Veterinary Medicine approved this study design.

### Samples and DNA processing

**Figure-1:**
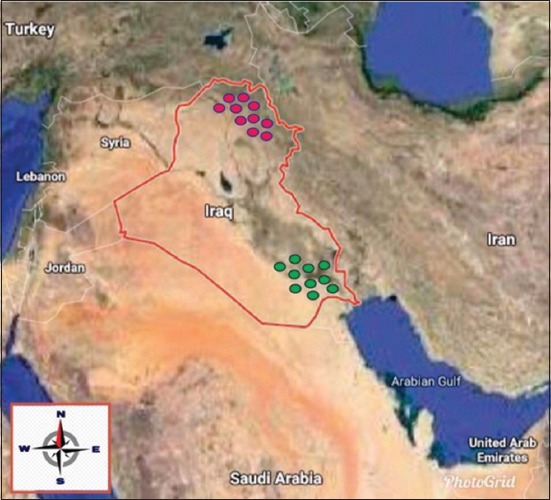
Global position system map of Iraq depicts the study area. Red cycles represent the samples from the Northern territory while green cycles refer to the samples from the South region.

The necropsied tracheal tissue from layer chickens (n=15) was collected from private laboratories in two different regions in Iraq (the North territory and the South region) (Figure-1). This is because both regions have significantly different farming systems and vaccination programs. The tissue samples were homogenized by ceramic beads (Thomas Scientific, USA) with phosphate-buffered saline (1 g/2 mL). Then, 500 µL of the homogenized tissues were centrifuged at 12.000*×* g for 1 min while the pellet (by QIAamp DNA tissue and blood kit) was used for DNA extraction according to the manufacturer’s recommendation.

### PCR

Analysis of ILTV sequences was conducted using PCR in a total reaction volume of 25 µL consisting of 12.5 µL of 2× Qiagen Multiplex Master Mix and includes Hot Start Taq DNA polymerase with a concentration of 3 mM Mg^2+^, as well as 1 µM of each, forward and reverse primers, in addition to 400 ng of template DNA (Table-1). The amplification process was conducted in a T100 thermal cycler (Bio-Rad, USA) with the temperature profile as follows: 95°C for 12 m then 45 cycles of 20 s at 95°C, followed by 30 s at 46°C (annealing), then 30 s extension at 72°C, followed by a final extension at 72°C for 10 m. All PCR amplicons were electrophoresed in agarose gel (1%) with 1× TAE buffer (40 mM Tris-base, 20 mM acetic acid, 1 mM EDTA) and visualized using SYBR^®^ Safe DNA Gel Stain (1×) (Invitrogen, UK) [12].

**Table-1:** Sequence of the primers used in the multilocus polymerase chain reaction.

Oligo name		Oligo sequence 5’→3’
UL54	F	CACAATGTTCTGGCCGGGTC
	R	GTGATGATATCGATCGTGATAGAG
UL52	F	CTATTACTGGCAACATGGCCTG
	R	CTTGGCTGACATGCTGGTCAG
gB	F	CTCTGGTGGCAAGTATCCTG
	R	CAGACGGTACTTTCTGTTGG
ICP18.5	F	CGATCGAAGAAGTTTTGTGCC
	R	GAGACGGCGAAAGCAAATGG
ICP4	F	CAAGTTTTTGCCATGGGGAC
	R	CATGACAGGCGCAAAAGAC
gJ	F	GAAACACACTTTTTCACTCAGGC
	R	CATGGAATTCTGAAACAACAGTAGG

F=Forward, R=Reverse

### DNA sequencing

All PCR amplicons were purified according to the QIAquick PCR Purification kit manual recommendations (Qiagen, UK) and measured according to the Invitrogen Qubit^®^ Fluorometer recommendations, then sequenced at Source Bioscience (Rochdale, UK) by Sanger Sequencing service. The generated sequences were aligned at BLASTn (http://www.ncbi.nlm.nih.gov/) to confirm the targeted sequencing. Thereafter, they were aligned by BioEdit software (Ibis Biosciences Inc., CA, USA) for pairwise alignment checking. Subsequently, the phylogenetic tree of each gene was generated using MEGA7 software, version 7.0.21(https://www.megasoftware.net/).

### Western immunoblotting

The Western immunoblotting served to explain the native molecular size of gE. First, whole protein lysates were extracted by cutting the tissues into small pieces (150 mg) then adding 500 µL of RIPA buffer (50 mM Tris, [pH 7.5], 150 mM NaCl, 1% NP40 alternative [v/v], 0.5% sodium deoxycholate [w/v], and 0.1% sodium dodecyl sulfate [SDS]) followed by adding 300 µL of a protease inhibitor cocktail (EDTA 100 mM, AEBSF 23 mM, Pepstatin A 0.3 mM, E-64 0.3 mM, and Bestatin 2 mM) (Sigma, USA). The lysates were sonicated with ultrasonic apparatus (VCX130, Sonic) for 7 s. The supernatants were collected, quantified using bicinchoninic acid assay (Pierce^™^ BCA Protein Assay Kit, UK) and heated for 5 min at 95°C. Thereafter, 20 µg of denatured protein samples were electrophoresed in SDS polyacrylamide gel electrophoresis (12%) with 5 µL of protein ladder (Spectra^™^, Thermo Scientific) using 1× electrophoresis running buffer, containing 192 mM glycine, and 50 mM Tris 0.1% (w/v) SDS pH 8.3, in a vertical gel electrophoresis tank (ATTO, Japan) at 200 V for 2 h at room temperature (23°C) [13]. The wet transfer method was used to transfer the separated proteins onto the polyvinylidene fluoride (PVDF) membrane (Merck, UK) at 200 V and 350 mA for 1 h. The transferred proteins were then blocked by skimmed milk (5%) (marvel, UK) that was dissolved in 1× Tris buffer saline-tween 20 (150 mM NaCl, 20 mM Tris-HCl, and 0.1% [v/v] tween 20, pH 7.5) for 12 h at 4°C with slow rotation. Thereafter, the PVDF membrane was subjected to the primary antibody (rabbit polyclonal antibody to ILTV glycoprotein E) at 1:2000 concentrations for 2 h at room temperature (23°C) (Biorbyt, UK). The bound antibody was then incubated for 1 h with goat anti-rabbit immunoglobulin G (conjugated with horseradish peroxidase) at 1:10000 (Vector Laboratories, PI-1000). The unbound antibodies were washed 3 times with 1× Tris-buffered saline-T for 10 m at room temperature with shaking. Finally, protein bands were developed and detected by substrate enhanced chemiluminescence (0.1 mL/cm^2^) and visualized by ChemiDoc^™^ Touch Imaging Systems (Bio-Rad, USA) [14].

## Results

### PCR

**Figure-2:**
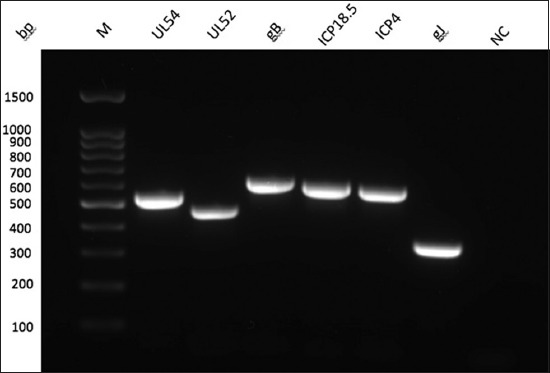
Gel electrophoresis (1%) image shows the amplified polymerase chain reaction products of the targeted genes of infectious laryngotracheitis virus including: UL54=544 bp, UL52=473 bp, gB=638 bp, ICP18.5=627 bp, ICP4=603 bp, gJ=367 bp and NC=Non-template control. M is the molecular weight marker (Cleaver Scientific, UK).

All the targeted genes including UL54, UL52, gB, ICP18.5, ICP4, and, gJ were successfully amplified at molecular sizes 544, 473, 638, 627, 603, and 367 bp, respectively, as shown in Figure-2. All the above-mentioned genes were sequenced and then aligned to generate the phylogenetic tree.

### Field and vaccine strains association

The results showed a considerable association within the ILTV strains in the two geographical regions of Iraq (North and South) as some samples exhibited a close phylogeny. More importantly, the results show a degree of relatedness of the field and vaccine strains across all genes (ICP18.5, UL54, ICP4, UL52, gB, and gJ). Some strains, originating from both geographical regions, revealed genetic interference with a locally used vaccine strain known as Laryngo-Vac (Zoetis Inc., USA).

**Figure-3:**
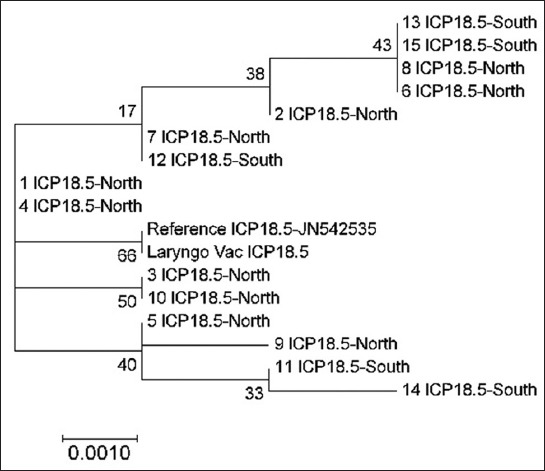
Phylogenetic tree of infectious laryngotracheitis virus field and vaccine genetic sequences for gene ICP18.5, compared with a reference gene (stated with accession number next the gene type) generated by maximum likelihood method from a nucleotide sequence alignment in MEGA7, with a bootstrap of 1000 replicates to provide support for individual nodes. The geographical origin (North or South) from the bird in which samples were taken from and the gene type is referred next to the sample name.

**Figure-4:**
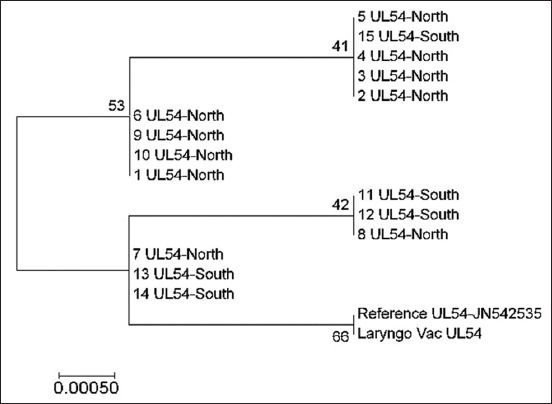
Phylogenetic tree of infectious laryngotracheitis virus field and vaccine genetic sequences for gene UL54, compared with a reference gene (stated with accession number next the gene type) generated by maximum likelihood method from a nucleotide sequence alignment in MEGA7, with a bootstrap of 1000 replicates to provide support for individual nodes. The geographical origin (North or South) from the bird in which samples were taken from and the gene type is referred next to the sample name.

Analysis of the ICP18.5 gene demonstrated a clear genetic interference between the sequences of the North and South regions. These sequences were not closely associated with the vaccine and reference strains (Figure-3). A similar finding was observed regarding UL54, as there was no significant genetic interference between the vaccine and field sequences. Nevertheless, a similar genetic association of the field samples from both North and South regions was shown in this targeted gene as all the sequences were clustered in four groups (Figure-4).

**Figure-5:**
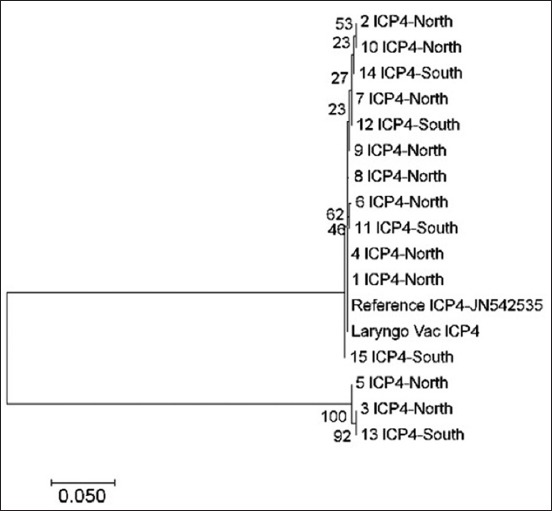
Phylogenetic tree of infectious laryngotracheitis virus field and vaccine genetic sequences for gene ICP4, compared with a reference gene (stated with accession number next the gene type) generated by maximum likelihood method from a nucleotide sequence alignment in MEGA7, with a bootstrap of 1000 replicates to provide support for individual nodes. The geographical origin (North or South) from the bird in which samples were taken from and the gene type is referred next to the sample name.

**Figure-6:**
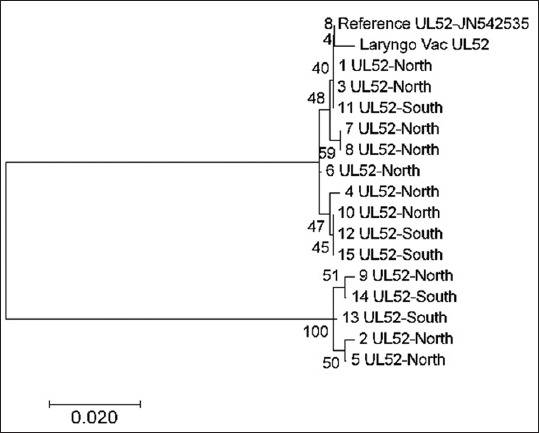
Phylogenetic tree of infectious laryngotracheitis virus field and vaccine genetic sequences for gene UL52, compared with a reference gene (stated with accession number next the gene type) generated by maximum likelihood method from a nucleotide sequence alignment in MEGA7, with a bootstrap of 1000 replicates to provide support for individual nodes. The geographical origin (North or South) from the bird in which samples were taken from and the gene type is referred next to the sample name.

In the ICP4 gene, all samples were genetically split into two groups; most of them were clustered within the vaccine and reference strains due to high genetic similarity (Figure-5). A similar phylogenetic association, but to a lesser extent, was seen in UL52 gene sequencing as most samples were clustered within three groups. In one group, including the vaccine and reference strains, most of the tested sequences originated from the samples from the North, except for one sample that originated from the South region. Only one sample (number 6) was disconnected from these groups of North origin (Figure-6).

**Figure-7:**
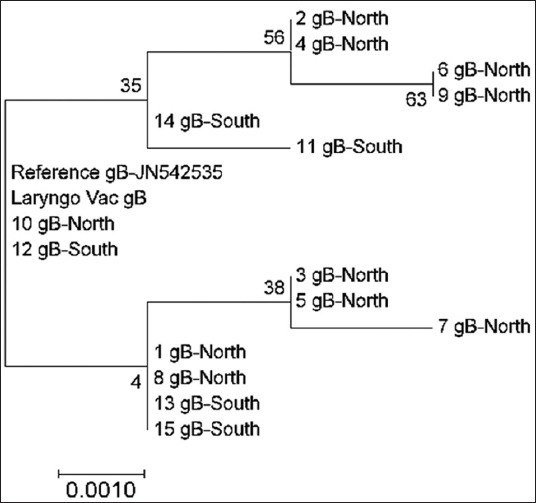
Phylogenetic tree of infectious laryngotracheitis virus field and vaccine genetic sequences for gene gB, compared with a reference gene (stated with accession number next the gene type) generated by maximum likelihood method from a nucleotide sequence alignment in MEGA7, with a bootstrap of 1000 replicates to provide support for individual nodes. The geographical origin (North or South) from the bird in which samples were taken from and the gene type is referred next to the sample name.

Analysis of gB gene sequences demonstrated a distinctive association between the field samples that originated from both North and South geographical regions. However, two samples from the South (sample numbers 13 and 15) revealed a similarity with two Northern strains (sample numbers 1 and 8). Similarly, sample numbers 14 and 11, from the Southern region, were branched from many of the Northern samples. Notably, the results exhibited a genetic interference between the vaccine strain and sample numbers 10 and 12, both of which originated from Northern and Southern regions (Figure-7).

**Figure-8:**
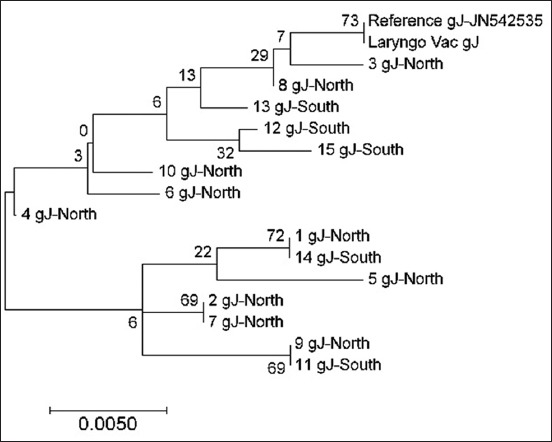
Phylogenetic tree of infectious laryngotracheitis virus field and vaccine genetic sequences for gene gJ, compared with a reference gene (stated with accession number next the gene type) generated by maximum likelihood method from a nucleotide sequence alignment in MEGA7, with a bootstrap of 1000 replicates to provide support for individual nodes. The geographical origin (North or South) from the bird in which samples were taken from and the gene type is referred next to the sample name.

In the gJ gene, a clustering pattern was noticed within the samples and where they originated from. The samples from the Northern region branched off and stayed separated from the Southern samples, with the exception of two samples from the South (sample numbers 11 and 14) (Figure-8). As observed in the gB gene, a similar genetic branching occurred in the gJ gene, demonstrating a genetic interference with the vaccine strain; however, this was only seen in one sample of Northern origin (sample number 3).

### Western immunoblotting

**Figure-9:**
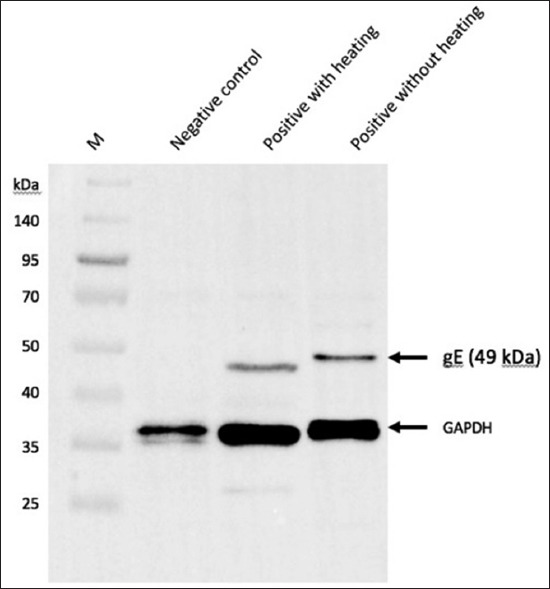
Western immunoblotting for glycoprotein E in two positive samples that showed a molecular weight of 49 kDa (1: Negative sample by polymerase chain reaction, 2: Positive sample which treated with heat, and 3: Positive sample without heat treatment). These were probed using rabbit polyclonal antibody to infectious laryngotracheitis virus glycoprotein E at 1:2000 dilution which consequently incubated with goat anti-rabbit immunoglobulin G (IgG) (conjugated with horseradish peroxidase [HRP]) at 1:10000. The glyceraldehyde 3-phosphate dehydrogenase was detected at 37 kDa using monoclonal antibody of mouse origin (0.5:20,000) (Sigma, UK) and HRP-conjugated anti-mouse IgG of horse origin (1:6000) (Sigma, UK). M is molecular weight marker (Spectra™ Broad Range Protein Ladder, Thermo Fisher Scientific, UK).

The native gE glycoprotein expression was observed to be approximately 49 kDa. However, the heat-denatured positive samples exhibited a slightly different result, with approximately 48 kDa in size due to the physical denaturation of the glycoprotein (Figure-9).

## Discussion

This study demonstrated that there is significant genetic variability between strains of ILTV. The results revealed a high level of relatedness between field outbreak strains and vaccine strains with different degrees of relatedness depending on the gene being assessed.

A vaccination program immunizing against ILTV has not officially been adopted by the federal government of Iraq [15] because of the political and economical disturbances. However, some farmers import the live-attenuated vaccine illegally from neighboring countries such as Syria, Turkey, Iran, Saudi Arabia, and Jordan (Figure-1). Therefore, ILTV outbreaks appear to evolve as a result of using the same attenuated vaccine strain throughout the geographical regions. The separation, or branching off, noticed in the Northern and Southern samples in the gJ gene exhibited more relatedness to the Northern field strain than with the vaccine strain. In particular, sample number 3, and the clustered grouping of ICP4 samples could imply that as the outbreak has advanced, the ILTV genome has changed. The branching of the ILTV samples originating from the Northern and Southern regions of Iraq may be caused by genetic recombination with the vaccine strain that has been used over extended periods of time. At some point, it is possible that genetic interference occurred between ILTV and VLT, giving later outbreak samples enough new genetic material to form a branch of their own; as a new strain. It is worth noting that both the Northern and Southern field samples across all genes result in phylogenetic trees that branch, which suggests that during the outbreak, this genetic variation of ILTV was carried across a body of water (possibly due to the trading of poultry between these areas). This finding is supported by research as it is possible for infected wild birds to shed ILTV particles thereby infecting birds in new areas, as well as the spreading of ILTV during transport of poultry and eggs [16].

It has been found that field strains of ILTV from Iraq are strongly related to vaccine strains, indicating that continued use of current attenuated vaccines leads to recombination and genetic diversity in Iraqi’s farms. Numerous studies have shown that most of the global outbreaks are the result of CEO vaccine recombination. For instance, the genetic analysis conducted by RFLP revealed potential similarities between filed strains of a Korean outbreak and CEO vaccine, indicating that vaccine recombination was responsible for the epidemic [4]. In Australia, there has been a new virulent ILTV formed through recombination of the indigenous vaccine, SA2 and A20, with the European Serva CEO vaccine, resulting in the replacement of the wild ILTV strain Class 2 [17]. In the same country, several genomic regions of ILTV including: gG, ICP18.5, ICP4, TK, gE, and B-TK were analyzed by PCR-RFLP which revealed the reversion effect of using attenuated vaccines in causing a disease outbreak [18].

The current issue results from developing and using a vaccine from a foreign strain. This is most likely what leads to the introduction of new ILTV genes into a region. For instance, a US CEO has been found to be the original wild ILTV recombination from a strain Italy [19]. All current ILTV strains published in GenBank have been compared with two new additional sequences from China and found that these strains did not represent a geographical phylogeny but rather formed clusters around vaccine use, suggesting that vaccine reversion plays an important role in current ILTV strains [20]. The current study could prove a suitable tool for assessing ILT and help decide where it is necessary to recruit stronger biosecurity.

The second phase of this study involved detecting the ILTV-gE from field strains to confirm the ILTV infection. This allowed us to distinguish between vaccinated and infected samples thus, testing only the infected samples by PCR for genetic analysis. Moreover, explaining at which molecular weight the native gE is expressed could be utilized to evaluate the possibility of using this glycoprotein as a vaccine candidate. Our results exhibited gE expression at 49 kDa. Eventually, this glycoprotein had been expressed [21] as a tag fusion protein with histidine and was then sized at 42.1 kDa. It was observed as an efficient antigen to detect and discriminate against the vaccinated flocks from unvaccinated ones. In this study, the size of gE is slightly higher than expected. This is likely due to the post-translational modification in the mammalian cells as this mechanism does not exist in prokaryotic cells [22]. To support this explanation, gE consists of 410 amino acids (accession number AFD36640.1), and theoretically, it might be expressed at 45.13 kDa (https://www.bioinformatics.org/sms/prot_mw.html). Similar findings have recently been observed in a study [23] where gE was expressed at 49 kDa in HEK 293 T cells by a mammalian expression system. However, a higher molecular weight was observed [3] at 75 kDa with N- glycosylation in chicken cells infected by ILTV.

As in other alpha herpesviruses, gE and gI are expressed as a heterodimer and have been found to have a pivotal role in cell-cell infection, while deleting this complex leads to loss of ILTV virulence [24].

## Conclusion

Our findings from this study may have an impact on vaccine production for ILTV prevention in the future. Utilizing the multilocus genetic profile of ILTV in the field strain could help epidemiologists and immunologists develop a more effective vaccine strategy. The current control approach for ILTV needs to be reassessed to avoid further outbreaks and spreading.

## Authors’ Contributions

MHAA designed and carried out the study. MHAA drafted, revised and approved the final manuscript.
